# Correlation Between Weber Classification of Ankle Fractures and Medial Clear Space Widening on Radiography

**DOI:** 10.3390/diagnostics15162085

**Published:** 2025-08-20

**Authors:** Philip Surmanowicz, Andrew Max Hamilton, Prosanta Mondal, Paul Kulyk, Navdeep Sahota, Haron Obaid

**Affiliations:** 1 Department of Medical Imaging, Royal University Hospital, College of Medicine, University of Saskatchewan, Saskatoon, SK S7N 0W8, Canada; jbq520@mail.usask.ca (P.S.); rbs680@mail.usask.ca (A.M.H.); nas229@mail.usask.ca (N.S.); 2 Clinical Research Support, College of Medicine, University of Saskatchewan, Saskatoon, SK S7N 5E5, Canada; prosanta.mondal@usask.ca; 3 Orthopedic Section, Department of Surgery, College of Medicine, University of Saskatchewan, Saskatoon, SK S7N 0W8, Canada; paul.kulyk@usask.ca

**Keywords:** ankle, fractures, weber, radiography

## Abstract

**Background/Objective**: There is a growing interest in deltoid ligament injury and repair. The integrity of the deltoid ligament is indirectly assessed through medial clear space widening. The objective of this study was to quantify the degree of medial clear space widening in Weber A, B, and C ankle fractures. **Methods**: Weber A, B, and C ankle fracture radiographs were retrospectively evaluated for the medial, lateral, and superior clear spaces and data gathered on associated injuries to the medial and posterior malleoli. Multivariable regression analysis was performed with the goal of assessing whether there were significant differences among the Weber fracture types for medial, lateral, and superior clear space widening. **Results**: A total of 473 radiographs with lateral malleolar fractures were retrospectively evaluated with 127 being Weber A, 216 Weber B, and 130 Weber C, with an additional 89 with associated fracture of the medial malleolus and 62 of the posterior malleolus. The mean medial clear space for Weber A fractures was 3.3 ± 1.1 mm, Weber B fractures 4.3 ± 2.4 mm, and Weber C fractures 5.7 ± 3.6 mm. Weber C fractures demonstrated significantly greater medial and lateral clear space distances than Weber A or B fractures. Additional fractures of the medial or posterior malleoli were also associated with greater medial and lateral clear space distances. **Conclusions**: Medial clear space is significantly increased in Weber C fractures and when additional medial or posterior malleolar fractures also occur. This sheds light on the biomechanics of ankle fractures and their impact on the medial ligamentous instability.

## 1. Introduction

Ankle fractures are one of the most common orthopedic injuries making up approximately 10% of all fractures [1] and 50% of all below-the-knee fractures [2]. These fractures often involve disruption to the deltoid or distal tibiofibular syndesmosis resulting in ankle instability. The deltoid ligament has been shown to be torn in 40% of ankle fractures when assessed arthroscopically [3] and 58% of ankle fractures when assessed with MRI [4]. While syndesmotic disruptions associated with fractures are commonly treated surgically, the deltoid ligament is not routinely repaired [5,6,7,8,9,10].

The deltoid ligament is composed of both superficial and deep layers forming a strong, triangular banded complex that plays a critical role in stabilizing the medial portion of the ankle. The superficial deltoid layer comprises the tibionavicular, tibiospring and tibiocalcaneal ligaments which originate at the medial malleolus and cross the ankle and subtalar joints, inserting onto the navicular bone, spring ligament and calcaneus. The deep deltoid layer comprises the anterior tibiotalar and deep posterior tibiotalar ligaments which originate at the medial malleolus and only cross the ankle joint, inserting into the talus [11,12]. The superficial layer restricts talar abduction, talar pronation and eversion of the hindfoot, while the deep layer primarily restrains the talus from external rotation [13,14,15]. Together the deltoid ligament layers act as a main stabilizer of the ankle preventing lateral and anterior displacement [16]. Multiple studies have shown that an intact deltoid ligament can stabilize the ankle mortise, despite lateral malleolar fractures or lateral ligamental injury [17,18,19,20,21].

Management of deltoid injuries is debated as there is no strong evidence demonstrating improved outcomes with surgical repair or possible indications for when repair should be considered [7,22,23,24,25]. Early studies reported that repair of the deltoid ligament was unnecessary following repair of the lateral malleolus and syndesmosis [5,6,9,10,26,27,28]. As such, most of the literature regarding ankle fractures has focused on lateral malleolar reduction and the tibiofibular syndesmosis. Recent studies however have shown repair of the deltoid ligament to be beneficial with improved stability and reduced complications [29,30,31,32,33,34,35,36]. Multiple meta-analyses have found that deltoid ligament repair may reduce post operative medial clear space widening as well as mal-reduction and reoperation rates [23,24,37], though significant heterogeneity amongst individual studies remains a concern. Another meta-analysis concluded deltoid repair may be warranted in patients with Weber C fractures or associated syndesmotic injury and fixation [22]. Overall, while more evidence is needed, there is growing interest in deltoid ligament injury and repair in the setting of ankle trauma.

Injury to the deltoid ligament is commonly assessed using ankle radiographs for widening of the medial clear space. The medial clear space is measured from the lateral border of the medial malleolus to the medial end of the talus with a medial clear space greater than 5 mm considered indicative of deep deltoid ligament rupture and instability [38]. Given the increased interest in deltoid ligament injury and repair, it would be of interest to know what patterns of lateral malleolar fractures result in greater medial clear space widening and in extension injury to the deltoid ligament. Therefore, the purpose of this study was to quantify the degree of medial clear space widening in Weber A, B, and C ankle fractures.

## 2. Materials and Methods

### Ethics

Approval from the University of Saskatchewan Institutional Review Board was obtained (Bio ID 4266), and in keeping with the policies for a retrospective review, informed consent was not required. Operational approval was also obtained from the health authority.

**Figure 1:**
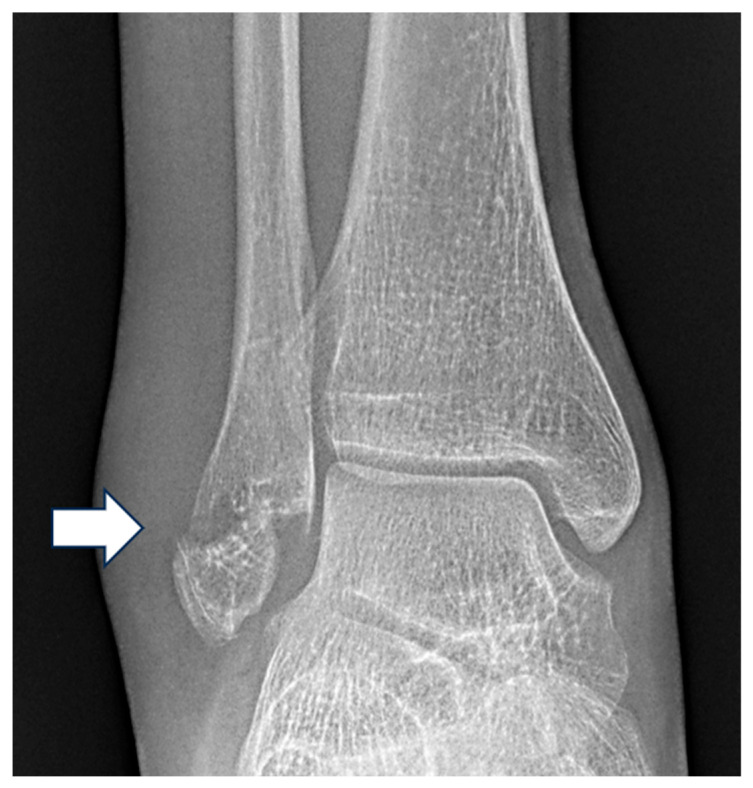
Mortise view radiograph of the right ankle demonstrating a displaced Weber A lateral malleolar fracture (white arrow) with normal lateral clear space, superior clear space and medial clear space.

**Figure 2:**
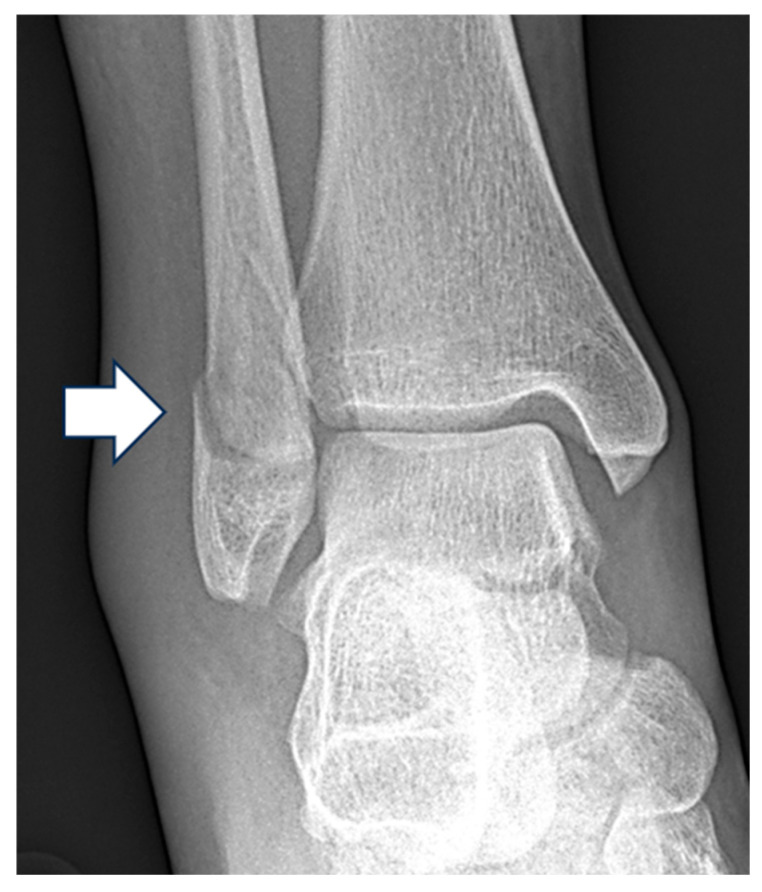
Mortise view radiograph of the right ankle demonstrating a displaced Weber B lateral malleolar fracture (white arrow) with normal lateral clear space, superior clear space and medial clear space.

**Figure 3:**
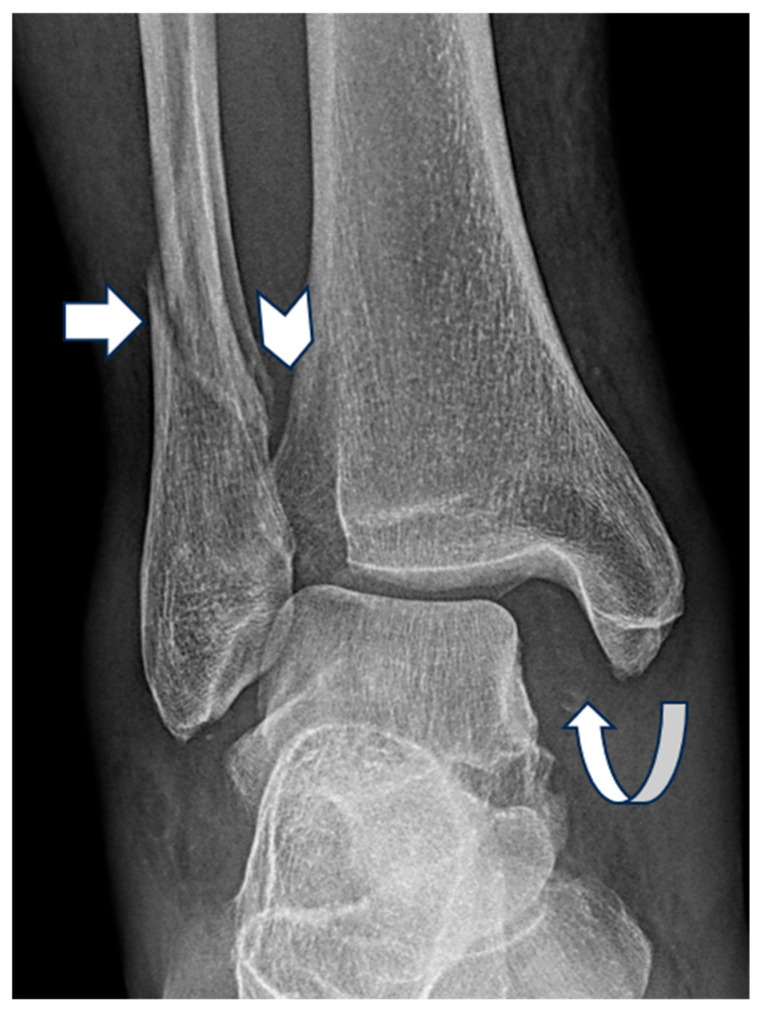
Mortise view radiograph of the right ankle demonstrating a displaced Weber C lateral malleolar fracture (white arrow) with widening of the medial clear space (curved arrow) and lateral clear space (chevron) but normal superior clear space.

A retrospective review of consecutive ankle radiographs with lateral malleolar fractures was performed. Subjects were selected using our electronic imaging database, Montage (Nuance^®^ mPower, Burlington, MA, USA). Keywords to select subjects with our database included “Weber A/B/C”, “Ankle fracture” and “lateral malleolar fractures”. Patients’ identifying information was kept anonymous using a master list and data collection tool. Inclusion criteria included patients greater than 18 years old, radiographs with a reported lateral malleolar fracture and radiographs performed with complete ankle series. Exclusion criteria included patients with ankle surgery, infection, tumors or incomplete radiographic series. In total, 473 patients with lateral malleolar radiographs were included in this study and stratified based on Weber ankle fracture classification (Figure 1, Figure 2 and Figure 3). Additional medial and posterior malleolar fractures were also noted.

**Figure 4:**
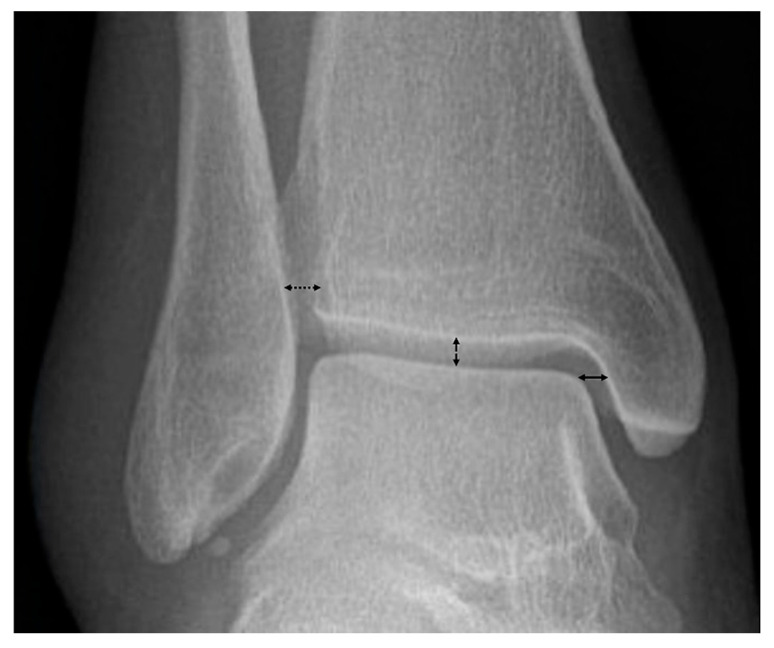
Medial, lateral, and superior clear space measurements. The medial clear space (solid line) was measured as the distance between the medial border of the talus and the lateral border of the medial malleolus at the level of the talar dome. The lateral or distal tibiofibular clear space (dotted line) is measured as the horizontal distance between the fibular notch and the medial edge of the distal fibula. The superior clear space (dashed line) was measured as the vertical distance from the highest point of the talar dome to the tibial plafond.

Measurement of the medial, lateral and superior clear space was performed. Measurements were performed on the mortise radiograph. The medial clear space was measured as the distance between the medial border of the talus and the lateral border of the medial malleolus at the level of the talar dome (Figure 4) [39,40]. The lateral clear space was measured as the horizontal distance between the fibular notch and the medial edge of the distal fibula (Figure 4) [39,41]. The superior clear space was measured as the vertical distance from the highest point of the talar dome to the tibial plafond (Figure 4) [42]. All images were reviewed on Picture Archiving and Communication System (Philips IntelliSpace PACS 4.4.541.5, Amsterdam, Netherlands) and displayed on Coronis Fusion 6-megapixel LED Barco monitors (MDCC-6230, Barco NV, Kortrijk, Belgium).

Analysis was performed by the local institutional Clinical Research Support Unit (CRSU) using Statistical Analysis Software, SAS/STAT^®^ version SAS 9.4 (SAS Institute Inc., SAS Campus Drive, Cary, NC 27513, USA.). Comparisons between categorical data were performed using a Chi-square test. Continuous data was compared using adjusted regression models. Statistical significance was set at *p* < 0.05.

## 3. Results

A total of 473 lateral malleolar fracture radiographs were assessed, of which 254 patients were female and 219 were male. The average patient age was 43.6 ± 19.1 years (mean ± SD). The distribution of lateral malleolar fractures included 127 Weber A, 216 Weber B, and 130 Weber C fractures. When comparing fracture patterns by gender, the distribution amongst females was 85 Weber A, 109 Weber B, and 60 Weber C. In comparison the distribution amongst males was 42 Weber A, 107 Weber B, and 70 Weber C. There was a significant difference in distribution with males trending towards more complex fractures (*p* = 0.002).

In addition to the lateral malleolar fractures, there was an associated medial malleolar fracture in 89 patients and an associated posterior malleolar fracture in 62 patients. The distribution of medial malleolar fractures was 13 with Weber A, 32 with Weber B, and 44 with Weber C. The distribution of posterior malleolar fractures was 6 with Weber A, 26 with Weber B and 30 with Weber C. Compared to those without an associated fracture, medial malleolar fractures (*p* < 0.0001) and posterior malleolar fracture (*p* < 0.0001) were significantly associated with more complex fractures (*p* < 0.0001).

The average lateral clear space distance amongst all fractures was 4.7 ± 2.1 mm. Average lateral clear space measured for Weber A fractures was 4.2 ± 1.4 mm, for Weber B fractures was 4.4 ± 1.5 mm and for Weber C fractures was 5.8 ± 2.9 mm (mean ± SD) (Table 1). Weber C fractures demonstrated a significantly greater lateral clear space compared to Weber A or B patterns (*p* < 0.0001). Fractures with an associated medial or posterior malleolar fracture demonstrated significantly greater lateral clear spaces as well (*p* = 0.0005; *p* = 0.0001, respectively).

The average medial clear space distance amongst all fractures was 4.5 ± 2.7 mm. Average medial clear space measured for Weber A fractures was 3.3 ± 0.7 mm, for Weber B fractures was 4.4 ± 2.4 mm and for Weber C fractures was 5.7 ± 3.6 mm (mean ± SD) (Table 1). Weber C fractures demonstrated a significantly greater medial clear space compared to Weber A or B patterns (*p* < 0.0001; 0.003, respectively). Fractures with an associated medial or posterior malleolar fracture demonstrated significantly greater medial clear spaces as well (*p* = 0.0009; *p* < 0.0001, respectively).

The average superior clear space distance amongst all fractures was 3.6 ± 0.8 mm. Average superior clear space measured for Weber A fractures was 3.4 ± 1.1 mm, for Weber B fractures was 3.6 ± 0.7 mm and for Weber C fractures was 3.7 ± 0.9 mm (mean ± SD) (Table 1). There was no significant difference in superior clear space between Weber fracture patterns. Fractures with an associated medial malleolar fracture demonstrated a significantly greater superior clear space (*p* = 0.009). There was no significant difference in superior clear space with an associated posterior malleolar fracture.

There was a significant negative correlation between age and lateral and medial clear spaces (*p* = <0.0001; 0.007, respectively. There was no correlation between age and superior clear space. Males were also found to have significantly larger medial (*p* = 0.01) and superior (*p* < 0.0001) clear spaces compared to females.

As the degree of medial clear space widening has been previously associated with deltoid injury, subjects were also stratified by medial clear space length into less than 4 mm, between 4 and 5 mm, and greater than 5 mm. These groups were chosen as 5 mm has been previously demonstrated as indicative of a deltoid injury [43,44,45]. The overall distribution of medial clear space included 219 less than 4 mm, 174 between 4 and 5 mm, and 79 greater than 5 mm. When comparing medial clear space by gender, the distribution amongst females was 147 less than 4 mm, 80 between 4 and 5 mm, and 27 greater than 5 mm. In comparison the distribution amongst males was 72 less than 4 mm, 94 between 4 and 5 mm, and 52 greater than 5 mm. There was a significant difference in distribution with males trending towards greater medial clear space widening (*p* = 0.002). There was no significant difference in lateral clear spaces between genders.

The medial clear space distribution amongst Weber A fractures was 90 (70.9%) less than 4 mm, 33 (26.0%) between 4 and 5 mm, and 4 (3.1%) greater than 5 mm. Amongst Weber B fractures, the distribution was 97 (44.9%) less than 4 mm, 83 (38.4%) between 4 and 5 mm, and 36 (16.7%) greater than 5 mm. For Weber C fractures, the distribution was 32 (24.8%) less than 4 mm, 58 (45.0%) between 4 and 5 mm, and 39 (30.2%) greater than 5 mm (Table 2). There was a significant difference in distribution with greater medial clear spaces trending towards more complex fractures (*p* < 0.0001).

Similar significant trends with greater medial clear spaces were seen with associated medial (*p* < 0.0001) and lateral (*p* < 0.0001) malleolar fractures. For lateral malleolar fractures without an associated medial malleolar fracture, the frequency of medial clear space widening included 196 (51.0%) less than 4 mm, 140 (36.5%) between 4 and 5 mm and 48 (12.5%) greater than 5 mm. For those with a medial malleolar fracture, there were 23 (26.2%) less than 4 mm, 34 (38.6%) between 4 and 5 mm and 31 (35.2%) greater than 5 mm. For lateral malleolar fractures without an associated posterior malleolar fracture, the frequency of medial clear space widening included 208 (50.7%) less than 4 mm, 152 (37.1%) between 4 and 5 mm and 50 (12.2%) greater than 5 mm. For those with a posterior malleolar fracture, there were 11 (17.7%) less than 4 mm, 22 (35.5%) between 4 and 5 mm and 29 (46.8%) greater than 5 mm.

## 4. Discussion

There is growing interest in injury and repair of the deltoid ligament due to recent studies showing potential benefits with repair following ankle fractures. Injury to the deltoid ligament is commonly assessed through the degree of medial clear space widening on ankle radiographs. This study sought to quantify the degree of medial clear space widening amongst different lateral malleolar fracture patterns to better understand which are most at risk of a deltoid ligament injury. The deltoid ligament comprises multiple superficial and deep components, and the combined strength is significantly greater than any of the lateral ligaments. Isolated deltoid ligament injuries are rare, occurring in 1–4% of all ankle ligament injuries [46,47,48]. Deltoid injuries are more commonly associated with lateral ankle injuries. Between 10 and 36% of deltoid injuries are associated with syndesmotic injuries [49,50,51], and 40% are associated with ankle fractures [3]. Our results support this, demonstrating that medial clear space is significantly increased in Weber C fractures, which are most commonly associated with lateral ligamentous injuries and additional ankle fractures [52,53,54]. We found additional medial or posterior malleolar fractures were most common in Weber C fractures and were both associated with increased medial clear space widening.

Medial clear space has been commonly used as a measure of deltoid injury. Prior studies have suggested that a medial clear space greater than 5 mm [38,55] or a medial clear space greater than 4 mm and 1 mm greater than the superior clear space [56,57,58] is indicative of a deep deltoid ligament injury. Our study found that Weber C fractures on average met these criteria with an average medial clear space of 5.7 mm and a superior clear space of 3.7 mm. Weber C fractures had a significantly higher frequency of medial clear spaces greater than 5 mm, affecting 30%. We report even higher frequencies of medial clear spaces greater than 5 mm with associated medial malleolar fractures, affecting 35% and associated posterior malleolar fractures, affecting 47%. These findings agree with prior studies where deltoid ligament rupture was most common in Weber C fractures [59]. With increased interest in deltoid ligament injury and repair, there may be benefits in focusing on Weber C fractures where medial clear space widening, instability, and deltoid injury are more frequent. One prior meta-analysis concluded that while further studies are needed, there may be benefit to deltoid ligament repair in Weber C fractures [22]. While there are false positives, these decrease as medial clear space increases greater than 5 mm [20].

Similarly to the medial clear space, we found the lateral or tibiofibular clear space significantly increased with Weber C fractures and associated medial and posterior malleolar fractures. The value of this measurement is debated [39,60], but measurements greater than 6 mm or an absence of tibiofibular overlap are considered abnormal and suggestive of syndesmotic injury [61,62]. We found an average lateral clear space of 5.8 mm in Weber C fractures suggestive of syndesmotic injury. Similarly, previous studies have reported syndesmotic injuries in 80% of Weber C fractures [54]. Both medial and lateral clear spaces were largest in more complex fractures which coincides with prior studies demonstrating deltoid injuries are often associated with syndesmotic injury [49,50,51].

Superior clear space did not significantly change between fracture patterns as demonstrated in prior studies [42,63]. This is due to the superior clear space assessing the distance of the talus and the tibia in the vertical plane. Unstable fractures, especially those with deltoid ligament injuries often involve lateral or external rotation of the talus [15,64,65,66], which is typically not reflected in the superior clear space. The superior clear space is more commonly used as a frame of reference to compare the medial clear space [42], with medial clear spaces greater than 4 mm and 1 mm greater than the superior clear space seen as suggestive of a deltoid injury.

We found greater medial clear space widening with ankle fractures in males with a higher frequency of medial clear spaces greater than 5 mm. This is likely secondary to males also experiencing more complex fractures with a higher frequency of Weber C fractures [67,68]. Previously, studies have similarly seen higher rates of Weber C fractures amongst males [69,70,71]. Our results further suggest males are more at risk of sustaining deltoid ligament injury and may benefit more from further evaluation of the deltoid ligament and possibly repair. This may be due to males being more likely to sustain ankle fractures from high-energy traumas [67], which are more prone to ligamentous injury and disruption of the ankle mortise [68]. Despite younger patients also experiencing a higher frequency of Weber C fractures [69,70], we did not see a higher frequency of medial clear spaces greater than 5 mm, though we did find a negative correlation between medial clear space and age. Prior MRI studies have found medial clear spaces greater than 5 mm were accurate for a deep deltoid ligament injury [68,70].

Limitations of this study include the retrospective nature of the study with potential selection bias; however, with the strict inclusion and exclusion criteria, we believe that this bias was minimized. We did not include gravity and manual stress radiographs in this study as these can be challenging to obtain with an acutely injured ankle and are not regularly obtained in the emergency departments at our institution. Similarly, we did not have MRI confirmation of deltoid ligament injury as not all patients with ankle trauma end up having an MRI. In addition, MRI studies have previously documented that medial clear spaces greater than 5 mm were accurate for a deep deltoid ligament injury.

## 5. Conclusions

In conclusion, there is growing interest regarding deltoid ligament injury and repair in the setting of ankle trauma. This study sought to quantify the degree of medial clear space widening in Weber A, B, and C ankle fractures to better understand which fracture patterns are more at risk of deltoid injury. Our results demonstrate that medial clear space is significantly widened in more complex Weber C fracture patterns with 30% having enough medial clear space widening to raise concern for deltoid injury. Further widening was seen with additional medial or posterior malleolar fractures with 35% and 46%, respectively, concerning for deltoid injury. While medial clear space is an indirect assessment of deltoid integrity, our results suggest patients with these fractures may benefit from further assessment of the deltoid such as stress imaging or surgical exploration.

## Figures and Tables

**Table 1:** Medial, lateral and superior clear space measurements by Weber fracture pattern.

Pattern (n)	Measurement	Mean ± Standard Deviation (mm)
Weber A (127)	Medial Clear Space	3.3 ± 1.1
	Lateral Clear Space	4.2 ± 1.4
	Superior Clear Space	3.4 ± 0.7
Weber B (216)	Medial Clear Space	4.4 ± 2.4
	Lateral Clear Space	4.4. ± 1.5
	Superior Clear Space	3.6 ± 0.7
Weber C (130)	Medial Clear Space	5.7 ± 3.6
	Lateral Clear Space	5.8 ± 2.9
	Superior Clear Space	3.7 ± 0.9

**Table 2:** Frequency of medial clear space widening by Weber fracture pattern.

Pattern (n)	Medial Clear Space	Frequency
Weber A (127)	<4 mm	90 (70.9%)
	4–5 mm	33 (26.0%)
	>5 mm	4 (3.1%)
Weber B (216)	<4 mm	97 (44.9%)
	4–5 mm	83 (38.4%)
	>5 mm	36 (16.7%)
Weber C (130)	<4 mm	32 (24.8%)
	4–5 mm	58 (45.0%)
	>5 mm	39 (30.2%)

## Data Availability

The data presented in this study are available on request from the corresponding author.
